# Intrinsic Capability of Budding Yeast Cofilin to Promote Turnover of Tropomyosin-Bound Actin Filaments

**DOI:** 10.1371/journal.pone.0003641

**Published:** 2008-11-04

**Authors:** Xiaoxue Fan, Skylar Martin-Brown, Laurence Florens, Rong Li

**Affiliations:** The Stowers Institute for Medical Research, Kansas City, Missouri, United States of America; University of Arkansas for Medical Sciences, United States of America

## Abstract

The ability of actin filaments to function in cell morphogenesis and motility is closely coupled to their dynamic properties. Yeast cells contain two prominent actin structures, cables and patches, both of which are rapidly assembled and disassembled. Although genetic studies have shown that rapid actin turnover in patches and cables depends on cofilin, how cofilin might control cable disassembly remains unclear, because tropomyosin, a component of actin cables, is thought to protect actin filaments against the depolymerizing activity of ADF/cofilin. We have identified cofilin as a yeast tropomyosin (Tpm1) binding protein through Tpm1 affinity column and mass spectrometry. Using a variety of assays, we show that yeast cofilin can efficiently depolymerize and sever yeast actin filaments decorated with either Tpm1 or mouse tropomyosins TM1 and TM4. Our results suggest that yeast cofilin has the intrinsic ability to promote actin cable turnover, and that the severing activity may rely on its ability to bind Tpm1.

## Introduction

The ability to assemble distinct and dynamic actin structures in the same cell is critical for the diverse role of actin in cell motility and morphogenesis [1]. In yeast cells that undergo polarized growth, actin cables and patches are dynamically assembled near growth sites and are required for two different cellular functions: polarized transport and endocytosis, respectively [2]. Actin filaments (F-actin) in cables are nucleated by formins and decorated by tropomyosins, whereas those in patches are nucleated by the Arp2/3 complex and decorated with cofilin and other patch specific markers [3]. Known cable components, Tpm1, Tpm2, Sac6, and Abp140, stabilize and/or bundle actin filaments *in vitro* and *in vivo* [6]–[11]. The rapid turnover of actin patches has been shown to depend on cofilin [4], and a recent study found that although cofilin was not detected along actin cables under normal conditions, rapid turnover of actin cables also requires cofilin [5].

Cofilin is a member of the ADF/cofilin family of low molecular weight actin-binding proteins. These proteins can bind to actin monomers (G-actin) and filaments and stimulate depolymerization and fragmentation of F-actin [12] **.** Every eukaryotic cell type examined to-date contains at least one member of the ADF/cofilin family, suggesting that cofilin performs a universally important function. Null mutations of cofilin in *Saccharomyces cerevisiae, Drosophila melanogaster* and *Caenorhabditis elegans* are all lethal. Cofilin-mediated F-actin severing and turnover are thought to be critical for rapid remodeling of actin organization in response to external stimuli, and for recycling of G-actin to sustain actin polymerization during cellular or intra cellular motility processes [13].

Tropomyosin has long been known to protect actin filaments from the depolymerizing and severing actions of DNaseI [14], ADF/cofilin [15], [16] and gelsolin [17]. Tropomyosin is a long protein with two α-helical polypeptides that form a parallel coiled coil, which further associate through head-to-tail interactions to form a long polymer along the side of an actin filament [18]. Whereas yeast has two tropomyosins, Tpm1 and Tpm2, vertebrates have 17 tropomyosin isoforms. In general, tropomyosin inhibits the dissociation of actin subunits from the pointed end of an actin filament [19], [20], which makes actin filament stronger and less likely to bend or break [21], [22]. Tropomyosin may also be a critical factor governing the spatial segregation of Arp2/3- and formin-nucleated actin structures in the cell. Whereas tropomyosin prevents branching nucleation by Arp2/3 complex, possibly due to their overlapping binding sites on F-actin [23], a recent study also suggests that tropomyosin actively facilitates assembly of actin filaments by formin protein [24].

In order to uncover the mechanism governing the disassembly of tropomyosin-bound actin filaments, we used a proteomics approach to identify tropomyosin-binding proteins and found cofilin to be a highly ranked tropomyosin binder. This led us to test if yeast cofilin can directly sever tropomyosin-decorated F-actin. Surprisingly, we found that the major yeast tropomyosin, Tpm1, has little protective effect against cofilin in a variety of assays, however, a cofilin mutant unable to bind Tpm1 showed diminished ability to sever Tpm1-bound F-actin. These results suggest that yeast cofilin is intrinsically capable of promoting the turnover of tropomyosin-bound F-actin in yeast.

## Results

Because previous studies suggest that tropomyosin inhibits actin depolymerization by cofilin [15], we hypothesized that additional proteins may be required to facilitate cable turnover, possibly through regulation of tropomyosin binding to actin, and set out to identify such proteins through Tpm1 affinity chromatography. For this and subsequent experiments, we used recombinant Tpm1 purified from *E. coli,* which we found had similar F-actin-binding properties as Tpm1 purified from yeast cells (data not shown). To minimize proteins that would associate with the Tpm1 affinity column via F-actin, yeast high-speed supernatant was prepared in G-actin buffer and indeed actin was not found among the bound proteins using Multidimensional Protein Identification Technology (MudPIT). Surprisingly, Cof1 was reproducibly detected in two Tmp1 affinity experiments, but not the BSA control columns, and was ranked among the top ten proteins, based on frequency of detection and quantitative NSAF values (Table 1).

**Table 1:** High-scoring binders of Tpm1 affinity purifications identified by MudPIT.

		Tmp1		BSA						
Rank^a^	Acronym	Urea^b^	KCl^b^	KCl^b^	Urea^b^	All_P^c^	All_sS^c^	All_uS^c^	All_SC^c^	All_NSAF^b^
1	Tpm1p	0.66589	0.30488	0	0.002	83	0	6159	100	0.14059
2	Ahp1p	0.00730	0.01830	0	0	10	0	333	81.82	0.00859
3	Tpi1p	0.01726	0.01236	0	0	16	0	416	74.6	0.00762
4	Eno1p	0.00438	0.01574	0	0	21	810	303	73.46	0.00629
5	Ipp1p	0.00282	0.00632	6.35E-5	0	13	0	221	61.67	0.00350
6	Pck1p	0.00052	0.00684	0	0	33	0	411	68.31	0.00340
7	Tsa1p	0.00267	0.00415	0	0	9	0	131	77.04	0.00304
8	Cof1p	0.00100	0.00372	0	0	9	0	101	73.43	0.00321
9	Tpm2p	0.00030	0.00952	0	0	10	0	85	68.94	0.00240
10	Yhr087wp	0.00171	0.00620	0	0	4	0	50	49.55	0.00205

a Proteins were first ranked based on their frequency of detection in the Tmp1 affinity purifications and their absence (or presence at much lower levels based on NSAF values) in the BSA negative controls. Proteins were then ranked based on their NSAF values averaged across the three Tmp1 experiments.

b NSAF (normalized spectral abundance factor) values measured in the Urea or KCl eluted samples and negative controls, as well as NSAF values calculated when spectral counts and proteins from all four runs were merged (ALL_NSAF).

c All_P, All_sS, and All_uS are respectively the peptide, shared spectral, and unique spectral counts when the four runs were merged, while All_SC is the final sequence coverage.

**Figure 1:**
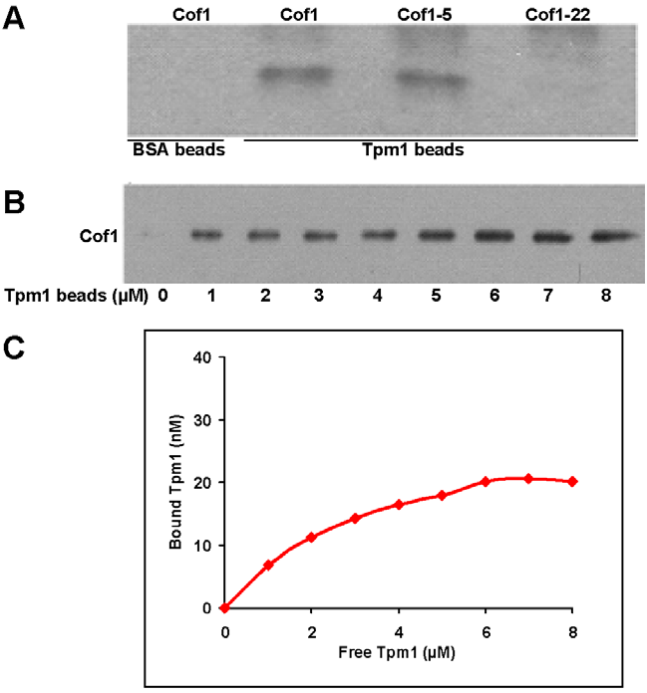
Yeast Cofilin binds Tpm1. A) Tpm1 binds directly to wild-type Cof1 and mutant Cof1-5 but not Cof1-22. BSA beads were used as a control. Beads coated with Tpm1 were incubated with 50 nM Cof1, Cof1-5 or Cof1-22. Bound cofilin was visualized by immunoblotting using yeast cofilin antibody [39]. B) Determination of dissociation constant K_d_ between Cof1 and Tpm1. Beads coated with 0 to 8 µM Tpm1 were incubated with 50 nM Cof1. Bound cofilin was visualized as in (A). C) Bound and free Tpm1 from (B) were quantified and plotted. The calculated K_d_ was 3.34±0.12 µM (mean±SD).

To test if the association between Cof1 and Tpm1 was direct, we expressed and purified yeast Cof1 from *E. coli* and found that it binds to Tpm1-coupled beads but not the control BSA beads (Fig. 1A). Using a quantitative Cof1 pull-down assay [25] the apparent K_d_ for Cof1 binding to Tpm1 was determined to be 3.34±0.12 µM (Fig. 1B). Despite a weak affinity, this interaction is likely to be specific because it was abolished by the *cof1-22* mutation, which was shown previously to inhibit turnover of actin patches and cables *in vivo* [4], [5], but not the *cof1-5* mutation (Fig. 1A). Neither mutation affected the folding stability of Cof1 [26].

**Figure 2:**
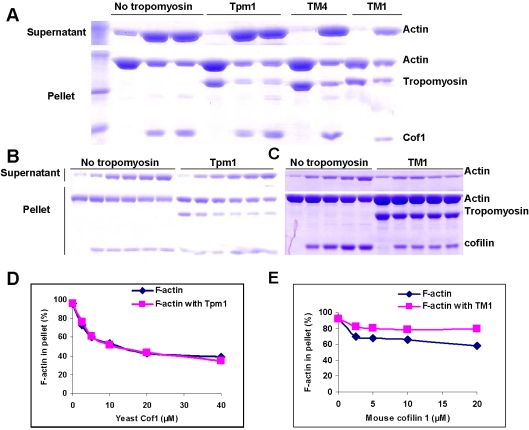
Yeast cofilin but not mouse cofilin 1 depolymerizes F-actin decorated with tropomyosin. A) Yeast F-actin (10 µM) polymerized with or without 10 µM tropomyosin was incubated with 0, 10 or 20 µM Cof1 (Tpm1-containing sample), or with 0 or 10 µM Cof1 (TM1 or TM4-containing sample). The supernatants and pellets after ultracentrifugation (see Materials and Methods) were analyzed on an SDS-PAGE gel. B) F-actin (10 µM) polymerized with or without 10 µM Tpm1p was incubated with 0, 2.5, 5, 10, 20 and 40 µM Cof1. Subsequent analysis was done as in (A). C) Rabbit muscle actin (10 µM) polymerized with or without 10 µM TM1 was incubated with 0, 5, 10, 20 and 40 µM mouse cofilin 1. Subsequent analysis was done as in (A). D, E) Quantification by densitometry of actin in pellet fractions (as % of the total actin) from experiments in (B) and (C), respectively.

**Figure 3:**
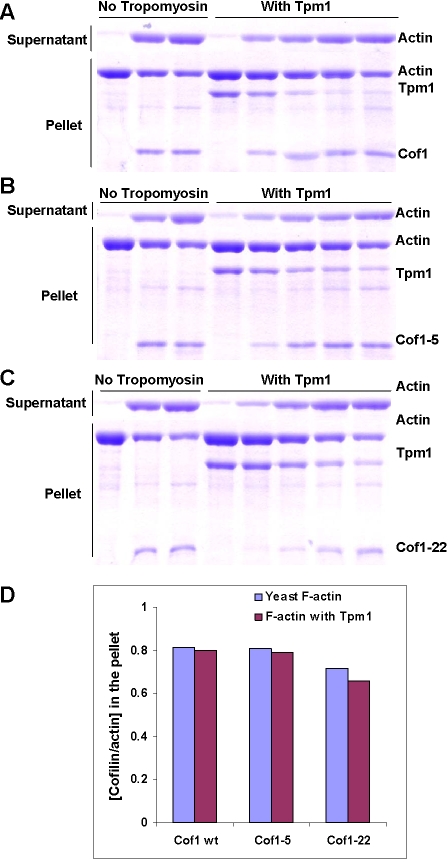
Yeast Cof1 mutants Cof1-5 and Cof1-22 can depolymerize F-actin with or without tropomyosin bound. A, B, C) F-actin (10 µM) polymerized without Tpm1p was incubated with 0, 10, 20 µM Cof1, Cof1-5 and Cof1-22 respectively and with 10 µM Tpm1p was incubated with 0, 2.5, 5, 10 and 20 µM Cof1, Cof1-5 and Cof1-22 respectively. The supernatants and pellets after ultracentrifugation (see Materials and Methods) were analyzed on an SDS-PAGE gel. D) The cofilin/actin molar ratios in the pellet from two independent co-sedimentation experiments using 10 µM actin and 10 µM cofilin. Ratios were determined by densitometry of Coomassie-stained SDS-gels shown in (A), (B) and (C).

The observation of a direct interaction between Cof1 and Tpm1 led us to test whether Cof1 impacts the stability of yeast F-actin decorated with Tpm1 using a co-sedimentation assay. Yeast actin, purified as described [27], was polymerized in the presence of Tpm1 and then incubated with or without an equimolar or excess cofilin for 20 min. In the absence of Cof1, Tpm1 co-sedimented with F-actin in the pellet fraction (Fig. 2A). Upon incubation with Cof1, most Tpm1 no longer sedimented in the pellet, but instead moved with F-actin to the supernatant fraction. Cof1 had a similar effect when incubated with yeast F-actin bound to either the mouse high-molecular-weight tropomyosin, TM1, or low-molecular-weight tropomyosin, TM4. The extents to which actin was solublized by cofilin were identical with or without tropomyosin bound. This is best observed in an experiment where increasing concentrations of Cof1 was used (Fig. 2B). The two yeast cofilin mutants, Cof1-5 and Cof1-22, showed a similar effect in this assay to that of wild-type Cof1 (Fig. 3). In contrast, mouse non-muscle cofilin 1, which is more similar than mouse cofilin 2 to yeast cofilin, showed diminished ability to depolymerize muscle actin bound to TM1 (Fig. 2C,E), TM4 or Tpm1, as expected (Table 2). This result raised the question of whether tropomyosin could indeed protect F-actin against yeast cofilin.

**Table 2:** Summary of cofilin depolymerization activity toward tropomyosin decorated actin filaments.

	Yeast Cof1	Mouse Cofilin 1
Yeast **F-actin**	**+ + + +**	**+**
Yeast **F-actin** with yeast **Tpm1**	**+ + + +**	**+**
Yeast **F-actin** with mouse**TM4**	**+ + + +**	**+**
Yeast **F-actin** with mouse **TM1**	**+ + + +**	**+**
Muscle **F-actin**	**+**	**+ + +**
Muscle **F-actin** with yeast **Tpm1**	**+**	**+ +**
Muscle **F-actin** with mouse **TM4**	**+**	**+ +**
Muscle **F-actin** with mouse **TM1**	**+**	**+ +**

Various sources of actin, cofilin and tropomyosin were tested using the co-sedimentation assay as in Figure 2 legend. ‘+’ refers to cofilin co-sedimenting with F-actin without causing depolymerization; ‘+ +’, ‘+ + +’ and ‘+ + + +’ refer to weak, moderate and strong, respectively, depolymerization of F-actin by cofilin.

**Figure 4:**
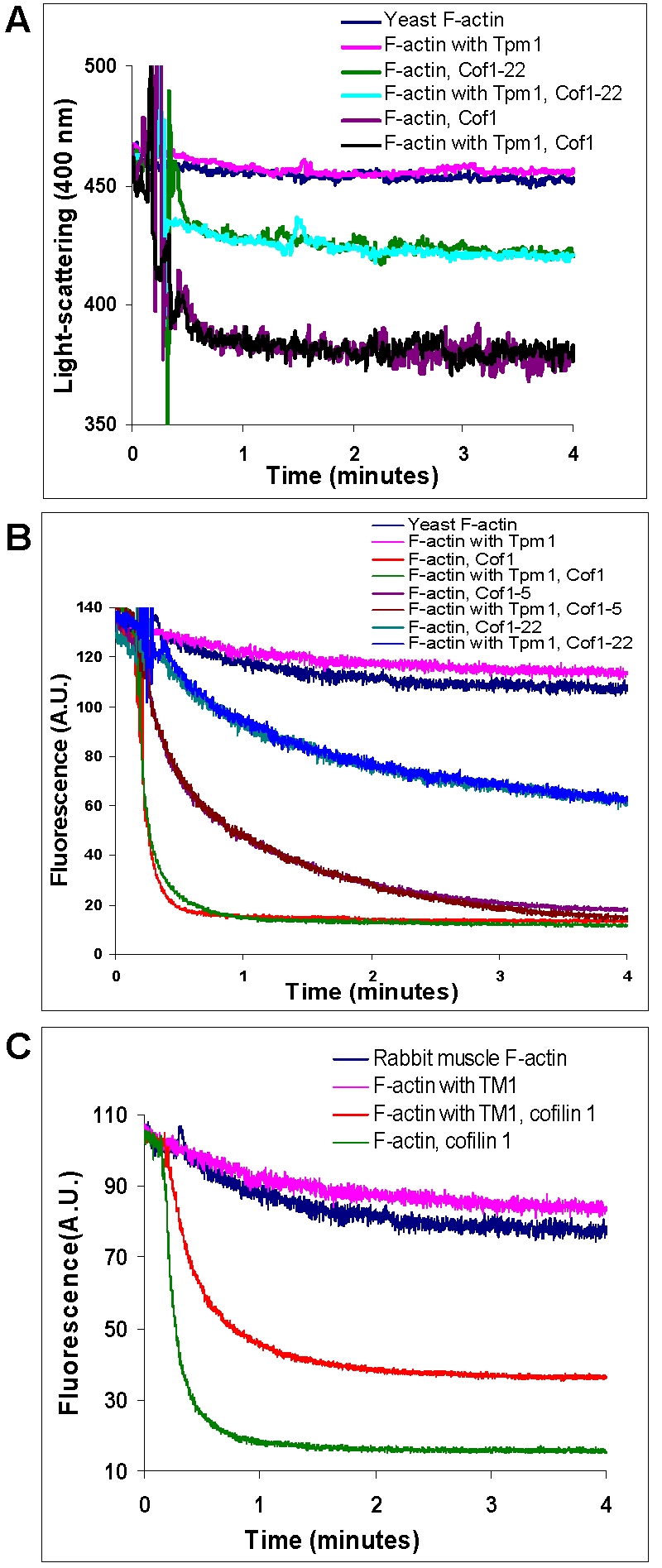
Effects of tropomyosin on actin depolymerization and actin binding by yeast Cof1 or mouse cofilin 1. A) Actin filament depolymerization was followed for 4 min at 25°C by the decrease in light-scattering at 400 nm. 5 µM yeast F-actin assembled in the presence or absence of Tpm1 was diluted to reactions containing final concentrations of 0.5 µM Cof1 or Cof1-22 and 0.5 µM F-actin. The spontaneous depolymerization of F-actin (no cofilin), with or without Tpm1 bound was monitored in parallel. B) Decrease in pyrene actin fluorescence was followed for 4 min at 25°C after dilution of F-actin (5 µM with 8% pyrene-labelled, assembled in the presence or absence of Tpm1) to a final concentration 0.5 µM F-actin with or without of 0.5 µM Cof1, Cof1-5 or Cof1-22. C) The same experiment as in (B) was carried out with muscle F-actin assembled with or without TM1, and in the presence or absence of mouse cofilin 1.

Because the above assay only assessed the steady-state distribution of F- versus G-actin, we next used two assays in which pyrene-labeled F-actin was diluted (see Materials and Methods) to determine the effect of Cof1 on the kinetics of actin depolymerization with or without bound tropomyosin. Light scattering showed that Cof1 drastically increased the rate of F-actin depolymerization after dilution, and Tpm1 binding to F-actin had no effect on the Cof1-induced actin depolymerization (Fig. 4A). Interestingly, measurements of pyrene actin fluorescence in these experiments suggest that binding of Cof1 to F-actin, as indicated by fluorescence quenching [28], was not affected by the presence of Tpm1 (Fig. 4B), On the other hand, mouse tropomyosin 1 reduced the binding of mouse cofilin 1 to F-actin (Fig. 4C), which is consistent with competitive binding of tropomyosin and cofilin to F-actin, as demonstrated by previous studies [16]. Cof1-22 exhibited reduced ability to promote depolymerization of naked actin compared to wild-type Cof1, confirming a previous report [4], yet again, Tpm1 did not alter the depolymerization kinetics in the presence of Cof1-22 (Fig. 4A).

**Figure 5:**
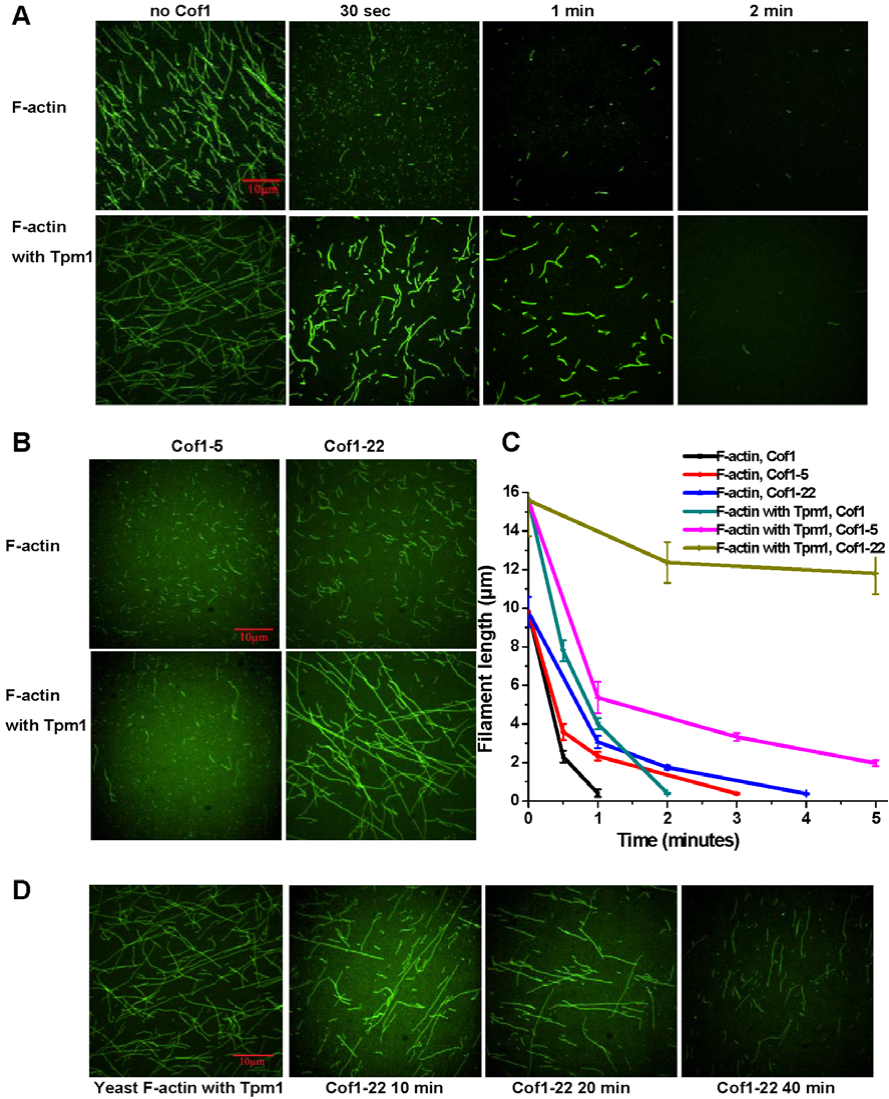
Severing of Tpm1-bound yeast F-actin by Cof1 but not Cof1-22. A) Representative confocal images of F-actin (5 µM), assembled without (upper panels) or with Tpm1 (lower panels), after incubation with 50 nM Cof1 for lengths of time as indicated (see Materials and Methods for more details). B) Representative confocal images of F-actin (5 µM), assembled without (upper panels) or with 5 µM Tpm1 (lower panels), after incubation with 50 nM Cof1-5 (left panels) or Cof1-22 (right panels) for 2 min. C) Measurements of actin filaments length from images recorded in experiments in (A) and (B). Shown are averages of filament length measurements from three fields per sample with error bars representing standard deviations. D) Representative confocal images of F-actin (5 µM), assembled with 5 µM Tpm1, after incubation with 50 nM Cof1-22 for lengths of time as indicated.

Cofilin possesses both actin depolymerization and severing activities, and the latter requires much lower concentrations of cofilin than the former [29]. We used a microscopy-based assay to test the effect of Tpm1 on actin severing by substoichiometric amounts of Cof1 (see Materials and Methods). Pre-polymerized yeast actin was incubated with Cof1 for various lengths of time. The reactions were stopped by dilution into a buffer containing Alexa488-phalloidin, and the lengths of F-actin were quantified using fluorescence microscopy. Under our experimental conditions, Tpm1 increased the average F-actin length from 9.81±4.56 µm to 15.6±3.45 µm (>100 filaments measured). After addition of 50 nM cofilin to 5 µM F-actin, the average F-actin length decreased rapidly in the presence or absence of Tpm1 (Fig. 5A). Even though Tpm1-bound filaments started longer, the rate of filament severing by Cof1 was similar with or without Tpm1 bound to F-actin. Cof1-5 exhibited an only slightly reduced rate of actin severing, which was not affected by Tpm1 binding (Fig. 5B,C). Interestingly, Cof1-22 only exhibited a moderate defect severing naked F-actin, however, the severing defect was much more enhanced toward Tpm1-bound F-actin, and long filaments were observable even after 40 min of incubation with Cof1-22 (Fig. 5B,C,D).

## Discussion

Tropomyosin-containing actin structures carry out many important functions such as cellular contractility, motility and polarized transport [18]. Current thinking on the turnover of tropomyosin-decorated actin structures is influenced by the notion that tropomyosin protects F-actin from the depolymerizing activity of ADF/cofilin. The data presented above show that tropomyosin does not present a barrier for the action of yeast cofilin, suggesting that Cof1 may be intrinsically capable of mediating the turnover of actin cables. This is consistent with the recent observation of a dramatically reduced rate of cable turnover in *cof1* mutant yeast cells, as well as in cells lacking Aip1, an *in vivo* co-factor that enhances ADF/cofilin-mediated actin fragmentation by capping the barbed ends of severed filaments [5].

The protective effect of tropomyosin against vertebrate ADF/cofilin is thought to be due to competitive binding of tropomyosin and ADF/cofilin to F-actin. We found surprisingly that Cof1 was associated as a high-scoring binder to Tpm1 affinity column, and that purified Cof1 binds directly to tropomyosin in solution. Although the affinity of this interaction is low in solution, it may occur more extensively on the surface of actin cables where there is a high local concentration of tropomyosin. The interaction between Cof1 and Tpm1 is abolished by *cof1-22*, which encompasses three amino acid substitutions: E134A, R135A, R138A [26]. These mutations were previously shown to reduce the binding of Cof1 to F-actin, and recent structural analysis placed these residues at the F-actin binding interface [30]. Based on these findings, we speculate that whereas the presence of Tpm1 may hinder Cof1's contacts with F-actin, some of these contacts may be replaced by the interaction between Tpm1 and Cof1. The latter could either alter the association of Tpm1 with F-actin or could help stabilize the overall interaction of Cof1 with F-actin in order to cause the structural changes in the filament that lead to fragmentation [31].

A previous study found that *cof1-22* also reduces the rate of actin patch turnover, and the extent of this reduction parallels the effect of the mutations on Cof1-induced actin depolymerization *in vitro* [4]. This is consistent with the high local concentration of Cof1 in actin patches and a specific requirement for Cof1 in the turnover of ADP-actin filaments during the endocytic process [32]. The effect of *cof1-22* on the rate of cable turnover, reported recently, appears much more dramatic than that on actin patches [5], and this correlates with the strong defect of the mutant cofilin in severing tropomyosin-decorated actin filaments observed in this study. These observations, together with a lack of localization of Cof1 along actin cables, may suggest that Cof1 promotes cable turnover primarily through its severing activity. Multicellular organisms express many isoforms of ADF/cofilin and tropomyosin due to gene duplication and alternative splicing [13], [18]. Even though some tropomyosin isoforms have been shown to antagonize the activity of ADF/cofilin, certain isoforms appear to actively recruit ADF/cofilin to highly dynamic actin structures [18]. Thus our results further support the view that cofilin and tropomyosin can collaborate in the regulation of rapid actin turnover.

## Materials and Methods

### Protein expression and purification

Genes encoding *S. cerevisiae TPM1*, *COF1*, mouse tropomyosin 1 (TM1) and 4 (TM4), and non-muscle cofilin 1 were amplified from yeast genomic DNA and mouse kidney cDNA library, respectively, and subcloned into pGEX-6P-1 vector (GE Healthcare) and expressed in *E. coli* BL21 cells as a glutathione S-transferense (GST) fusion proteins. Mutations corresponding to *cof1-5* and *cof1-22* [26] were introduced to Cof1 expression plasmid by site-directed mutagenesis. Recombinant proteins were cleaved from GST by PreScission protease and purified by passing through glutathione-agarose columns and by ion exchange chromatography (BioRad Econo-Pac Q). Native Tpm1 was purified from yeast Δ*tpm2* strain as previously described [33].

### Tpm1 affinity chromatography and MudPIT analysis

Purified Tpm1 (300 mg) were coupled to 5 ml active agarose (BioRad, Affi-Gel 10). Using HEK buffer (20 mM Hepes, pH 7.5, 1 mM EDTA, 50 mM KCl) supplemented with 10% glycerol and with 1 mM PMSF plus an aqueous cocktail of protease inhibitors, a high-speed supernatant was generated from lysed yeast cells (S288c), as described previously [34]. These yeast whole extracts were then loaded onto Tpm1-beads for over three hours and the column was washed overnight with HEK buffer. The specific Tpm1 binding proteins were eluted using different buffers: 0.5 M KCl or 3 M urea in HEK buffer. Negative controls using BSA-conjugated columns were also performed in parallel and proteins were eluted in 0.5 M KCl in HEK buffer or 3 M Urea in HEK buffer. TCA-precipitated proteins were urea-denatured, reduced, alkylated and digested with endoproteinase Lys-C (Roche) followed by modified trypsin (Roche) as described [35]. Peptide mixtures were loaded onto 100 µm fused silica microcapillary columns and placed in-line with a quaternary Agilent 1100 series HPLC pump and LTQ linear ion trap mass spectrometers (ThermoScientific). Fully automated 10-step MudPIT runs were carried out on the electrosprayed peptides, as described in reference [35]. Tandem mass (MS/MS) spectra were interpreted using SEQUEST [36] against a database of 11982 amino acid sequences, consisting of 5815 *Saccharomyces cerevisiae* proteins (non-redundant entries from NCBI 2007-03-12 release), 176 usual contaminants such as human keratins, IgGs, and proteolytic enzymes; and, to estimate false discovery rates (FDR), 5991 randomized sequences for each non-redundant protein entry. Peptide/spectrum matches were sorted and selected using DTASelect [37] and peptide hits from multiple runs were compared using CONTRAST [37]. Under the selection criteria we applied, FDR at the protein level was 1.85%, while the average spectral FDR was 0.4%. To estimate relative protein levels, Normalized Spectral Abundance Factors (NSAFs) were calculated for each detected protein, as described [38].

### Determining the binding affinity between Tpm1 and Cof1

K_d_ determinations by Tpm1-Gel 10 pull-down of purified Cof1 were performed as described [25]. Briefly Tpm1-coated Affi-Gel beads (0–8 µM) were incubated with 50 nM purified Cof1 and 2 mg/ml bovine serum albumin. The samples were incubated for 1 hr at 4°C with agitation. Supernatants were collected by low speed centrifugation. Beads were washed four times in 50 mM Tris-HCl, pH7.5, 150 mM NaCl, 1 mM EDTA, and 1 mM DTT with 1% Nonidet P-40. Free and bound fractions were detected by Western blotting for anti-Cof1 [39], after resolving the proteins by SDS-PAGE. The bands were quantified using ImageQuant software (Amersham Biosciences). The data were plotted and fitted with a single rectangular hyperbola equation: *B* = *B*_max_ *C*/(*K_d_*+*C*) using SigmaPlot software (Systat Software, Inc.).

### Actin cosedimentation assays

Yeast actin and rabbit skeletal muscle actin were purified using previously described procedures [27], [40]. The purified yeast or rabbit muscle actin (10 µM) was polymerized by adding 50 mM KCl, 5 mM MgCl_2,_ and 0.5 mM ATP in G-buffer for 2 hr at room temperature with or without 10 µM tropomyosins. Yeast Cof1 or mouse cofilin1 was incubated at specified concentrations with polymerized actin for 20 min at room temperature. The samples were centrifuged in a Beckman Optima TLX ultracentrifuge at 900,000 rpm for 20 min. The pellets were dissolved by protein gel loading buffer at an equal volume as the supernatant. The same volume of supernatants and pellets were analyzed by SDS-PAGE.

### Actin depolymerization assays

Yeast actin 5 µM was co-polymerized with 8% pyrene-labeled rabbit muscle actin at 25°C for 2 hr in F-buffer. F-actin and cofilins were mixed in a cuvette to final concentrations of 0.5 µM, and the decrease in pyrene fluorescence was monitored at 25°C in a fluorescence spectrophotometer (Photon Technology International) with an excitation wavelength of 365 nm and emission of 407 nm. In light-scattering assay, the depolymerization of F-actin was monitored at 25°C with both the excitation and emission wavelengths of 400 nm.

### Quantification of actin filament length by confocal imaging

Yeast actin of 5 µM was polymerized with or without 5 µM tropomyosin overnight at 4°C. Purified 50 nM Cof1 was added to and incubated with the preassembled F-actin for various lengths of time and then diluted and stained by Alexa-fluor 488 phalloidin. Actin filaments were imaged on a Zeiss Axiovert 200M with a Yokogawa spinning disc confocal microscope using a 100X DIC oil-immersion objective. Image acquisition was performed using a Hamamatsu EM-CCD camera. Actin filament lengths were quantified using the Metamorph image software.
